# Spinal instability in ankylosing spondylitis

**DOI:** 10.4103/0019-5413.65151

**Published:** 2010

**Authors:** Siddharth A Badve, Shekhar Y Bhojraj, Abhay M Nene, Raghuprasad Varma, Sheetal Mohite, Sameer Kalkotwar, Ankur Gupta

**Affiliations:** Department Of Orthopaedics, T. N. Medical College and B.Y.L. Nair Hospital, Research Fellow, Spine Foundation, Mumbai, India; 1 Spine Clinic, Lilavati Hospital and Breach Candy Hospital, Mumbai, India; 2 P.D. Hinduja Hospital and Research Centre, Mumbai, India; 3 L.H. Hiranandani Hospital, Mumbai, India; 4 Spine Clinic, Lilavati Hospital, Mumbai, India

**Keywords:** Ankylosing spondylitis, low energy fracture, spinal instability

## Abstract

**Background:**

Unstable spinal lesions in patients with ankylosing spondylitis are common and have a high incidence of associated neurological deficit. The evolution and presentation of these lesions is unclear and the management strategies can be confusing. We present retrospective analysis of the cases of ankylosing spondylitis developing spinal instability either due to spondylodiscitis or fractures for mechanisms of injury, presentations, management strategies and outcome.

**Materials and Methods:**

In a retrospective analysis of 16 cases of ankylosing spondylitis, treated surgically for unstable spinal lesions over a period of 12 years (1995-2007); 87.5% (n=14) patients had low energy (no obvious/trivial) trauma while 12.5% (n=2) patients sustained high energy trauma. The most common presentation was pain associated with neurological deficit. The surgical indications included neurological deficit, chronic pain due to instability and progressive deformity. All patients were treated surgically with anterior surgery in 18.8% (n=3) patients, posterior in 56.2% (n=9) patients and combined approach in 25% (n=4) patients. Instrumented fusion was carried out in 87.5% (n=14) patients. Average surgical duration was 3.84 (Range 2-7.5) hours, blood loss 765.6 (± 472.5) ml and follow-up 54.5 (Range 18-54) months. The patients were evaluated for pain score, Frankel neurological grading, deformity progression and radiological fusion. One patient died of medical complications a week following surgery.

**Results:**

Intra-operative adverse events like dural tears and inadequate deformity correction occurred in 18.7% (n=3) patients (Cases 6, 7 and 8) which could be managed conservatively. There was a significant improvement in the Visual analogue score for pain from a pre-surgical median of 8 to post-surgical median of 2 (*P*=0.001), while the neurological status improved in 90% (n=9) patients among those with preoperative neurological deficit who could be followed-up (n =10). Frankel grading improved from C to E in 31.25% (n=5) patients, D to E in 12.5% (n=2) and B to D in 12.5% (n=2), while it remained unchanged in the remaining - E in 31.25% (n=5), B in 6.25% (n=1) and D in 6.25% (n=1). Fusion occurred in 11 (68.7%) patients, while 12.5% (n=2) had pseudoarthrosis and 12.5% (n=2) patients had evidence of inadequate fusion. 68.7% (n=11) patients regained their pre-injury functional status, with no spine related complaints and 25% (n=4) patients had complaints like chronic back pain and deformity progression. In one patient (6.2%) who died of medical complications a week following surgery, the neurological function remained unchanged (Frankel grade D). Persistent back pain attributed to inadequate fusion/ pseudoarthrosis could be managed conservatively in 12.5% (n=2) patients. Progression of deformity and pain secondary to pseudoarthrosis, requiring revision surgery was noted in one patient (6.2%). One patient (6.2%) had no neurological recovery following the surgery and continued to have nonfunctional neurological status.

**Conclusion:**

In ankylosing spondylitis, the diagnosis of unstable spinal lesions needs high index of suspicion and extensive radiological evaluation Surgery is indicated if neurological deficit, two/three column injury, significant pain and progressive deformity are present. Long segment instrumentation and fusion is ideal.

## INTRODUCTION

Ankylosing spondylitis is a chronic inflammatory disorder belonging to the family of seronegative spondyloarthropathies. Ankylosing spondylitis has a prevalence of 0.1 to 1.4% correlating with the frequency of HLA B 27.^1^,^2^ This condition is usually diagnosed in the second or third decade of life, with a male preponderance in the ratio of 3:1.^3^ The disease predominantly involves the axial skeleton and is characterized by the ossification of intervertebral discs, joints and ligaments leading to progressive rigidity of the spine. Formation of syndesmophytes across the disc spaces affects the mobility of the entire spine resulting in a ‘bamboo spine’.

The initial presentation is with a low backache, due to early involvement of the sacroiliac joints and the lumbar spine, which eventually progresses to the cervical region.^4^ Progressive stiffening makes the spine less supple to ‘absorb’ the stress of sudden movement. Associated secondary osteoporosis due to immobility increases the fragility of the vertebral column. This makes the spine susceptible to vertebral fractures.^5^–^8^ Thus the risk of thoracolumbar fractures is four times that in the general population.^9^ Also, a significant proportion of these unstable spinal lesions can be attributed to no obvious trauma or trivial low velocity injuries.^6^,^7^,^10^–^15^ Spondylodiscitis, either due to inflammation or mechanical stresses, may play a vital role in the origin of such spontaneous unstable lesions which behave like stress fractures.^16^–^18^

The history of significant traumatic event is not elicited in most of these patients.^6^,^7^,^10^–^15^ The patients with ankylosing spondylitis, who suffer from continuous back pain, worsening of complaints may easily be (mis) interpreted as disease flare or muscle strain and the diagnosis gets delayed with consequent complications. Hence a high index of suspicion is necessary to make the diagnosis, especially when plain radiography is the screening modality. Management of these lesions can be challenging due to associated medical co-morbidities, anesthetic problems and numerous surgical difficulties which may lead to serious complications.^5^–^8^

In this retrospective analysis, we report the mechanics of these unstable spinal lesions, their presentation, diagnostic difficulties, strategies in the surgical management and the outcome in patients with ankylosing spondylitis.

## MATERIALS AND METHODS

We retrospectively analyzed 16 cases of ankylosing spondylitis, over a period of 12 years (1995-2007), presenting with spinal instability that was managed and followed up for mean 54.5 months (Range 18-154 months) [Table 1]. The study included 93.7% (15) males and 6.2% (one) female, with a (range of 30 to 75 years) a (mean age = 46.13 years). The mechanism of injury was identified as minor (low energy) in 87.5% (n=14) and major (high energy) 12.5% (n=2) patients. The patients in whom the injury was classified as minor had either a trivial fall or no history of any obvious injury; while those with major injury had a history of road traffic accident. The duration between the minor traumatic event and presentation to the physician was variable, usually a few months, and in some cases extending up to a few years. The delay in presentation could be attributed to factors like the pre-existing back pain and deformity, episodic worsening of pain due to disease flares, the injury being trivial or absent altogether and occult nature of the lesions. These factors made the accurate assessment of the duration between the ‘minor injury’ and presentation difficult. Depending on the spinal region involved, the injuries were classified as cervical, dorsal, lumbar or combined, when a concurrent lesion was present in more than one region.

**Table 1:** Clinical details of patients, presentation, management and follow-up

Pt. no.	Age/sex	Severity of trauma	Presentation	Level of fracture	Surgical procedure	Approach	Follow-up months	Pain status	Neurological status	Deformity	Fusion	Return to pre-injury function
1	50/M	Low energy	Neurodeficit instability	C6-7-T1	D + IF	Posterior	154	Improved	Improved	N A	Present	Present
2	55/M	Low energy	Neurodeficit instability	T10-11	D + F	Posterior	123	Improved	Improved	N A	Present	Present
3	32/M	High energy	Neurodeficit instability	C6-7	D + IF	Posterior	111	Improved	Same	N A	Present	Present
4	41/M	Low energy	Instability	T11-12	D + F	Anterior	72	Improved partially	N A	N A	Pseudo arthrosis	Absent
5	52/M	High energy	Neurodeficit instability	C5-6	D + IF	Anterior	63	Improved	Improved	N A	Present	Present
6	37/M	Low energy	Neurodeficit instability	C1-C2	D + IF	Posterior	39	Improved	Improved	Improved partially	Present	Present
7	60/M	Low energy	Neurodeficit instability	C5-6, L1-2	D + IF	Posterior	39	Improved	Improved	Improved	Present	Present
8	30/F	Low energy	Neurodeficit instability	T10-11	D + IF	Posterior	36	Deteriorated	Improved	Deteriorated	Pseudoarthrosis	Absent
9	42/M	Low energy	Instability	T12-L1	D + IF	Anterior	36	Improved	N A	N A	Present	Present
10	32/M	Low energy	Instability	T12-L1	D + IF	Combined (post-ant)	36	Improved	N A	Improved	Present	Present
11	36/M	Low energy	Neurodeficit instability	T11-12	D + IF	Combined (post-ant)	24	Improved	Improved	Improved	Present	Present
12	42/M	Low energy	Instability	L2	D + IF	Combined (post-ant)	22	Improved	N A	N A	Present	Present
13	41/M	Low energy	Instability	T11-12	D + IF	Posterior	22	Improved	N A	N A	Inadequate	Partial
14	75/M	Low energy	Neurodeficit instability	T11	D + IF	Posterior	22	Improved partially	Improved	NA	Inadequate	Absent
15	56/M	Low energy	Neurodeficit instability	T11-12	D + IF	Posterior	18	Improved	Improved	NA	Present	Present
16	57/M	Low energy	Neurodeficit instability	C5-6	D + IF	Combined (post-ant)			Same			Expired

NA = Not applicable; D = Decompression; IF = Instrumented fusion; F = Fusion

The presentation included persistent back pain, neurological deficit and progressive deformity either in isolation or combination. The pain was characterized by worsening of an already existing back pain. The duration of complaints before the presentation was variable. Spinal instability was defined on the basis of clinical and radiological features. The clinical features included a sustained elevation in the severity of chronic back pain that was further accentuated by loading the spine, in association with or without neurological deficit, leading to restriction of the activities of daily living and an increase in the requirement of analgesic dosage with localized spinal tenderness on examination. On the other hand, the radiological feature included a breach in the integrity of the spinal columns (two of three). Flexion and extension views were not attempted due to the apprehension of inducing or worsening of neurological deficit.

At the time of admission and follow-up, patients were assessed clinically and radiologically. In addition to noting the pain scores, a detailed clinical examination and neurological evaluation by using Frankel score was performed.^19^,^20^ The presence of associated co-morbid conditions was also noted. The radiological studies included plain radiographs, computed tomography (CT) and magnetic resonance imaging (MRI). Radiologically, the nature of the pathology, level, angle of kyphosis and integrity of the three columns were defined.^21^,^22^ The MRI and CT findings of a sclerotic lesion involving the three columns, absence of a predominant soft tissue component, evidence of pseudoarthrosis in an ankylosed spine aided in the diagnosis. On the other hand, findings like the extent of anterior/middle column defect, neurological compression and other morphological details of the lesion helped to plan the management.

The surgical indications included neurological deficit, evidence of spinal instability with progressive pain and deformity. The selection of the approach and the surgical procedure was based on the presentation of patient, neurological evaluation, the nature and location of the lesion, spinal alignment noted on clinical and radiological evaluation, intra-operative findings and the presence of co-morbid conditions. The surgical procedure in majority of the cases included decompression, stabilization and fusion with bone grafting. Deformity correction was attempted in a few selected cases with a severe progressive deformity. A combination of posterior and anterior approach was used in the presence of three-column instability with significant anterior/middle column defect and a kyphotic spine requiring posterior stabilization and anterior reconstruction. An exclusive posterior approach was used in patients with minimal kyphosis and no significant anterior/middle column defect, even after posterior stabilization. An exclusive anterior approach could be used in a few patients with significant anterior/middle column defect with minimal kyphosis and a relatively intact posterior column. A consideration to the regional distribution of the lesion was also given while deciding the approach. Anterior approach to dorsal and dorsolumbar regions could result in significant morbidity especially in patients with poor pulmonary function and this risk was given due consideration.

Majority (87.5%, n=14) of the patients were instrumented to augment the unstable spine, posterior stabilization being most common. A combined posterior and anterior instrumentation was used in highly unstable spinal lesions requiring additional stabilization. A pure anterior instrumentation was carried out either in the cervical region, where plating was convenient or in a lesion with predominant anterior defect with minimal kyphosis with an intact posterior column, in which case only an anterior surgery was considered sufficient. Instrumentation was avoided when the spinal stability was adequate or when instrumentation was not possible due to poor soft tissue cover. The surgical duration and the intra-operative blood loss were also noted.

The follow-up duration was calculated from the date of admission. The patients were evaluated clinically and radiologically every three months. The following criteria were used to assess the operative results. The severity of pain evaluation was based on Visual analogue scale while the neurological assessment was based on Frankel grading.

The deformity was assessed clinically and radiologically based on chin brow angle, wall occiput distance, finger floor distance and the angle of kyphotic deformity. The evaluation of radiological fusion was based on consolidation of the graft, healing at the interface with appearance of trabecular pattern across the fracture site, along with absence of implant failure/back out. A return to pre-injury functional status was also noted. Results were graded on the basis of improvement or maintenance of the above mentioned criteria at the time of follow-up in comparison to the pre-injury and early postoperative status.

## RESULTS

The mean age of patients was 46.1 (range 30-75 years). There were 25% (n=4) cervical, 43.8% (n=7) dorsal, 6.2% (n=1) lumbar, 6.2% (n=1) cevicodorsal, 12.5% (n=2) dorsolumbar along with 6.2% (n=1) combined cervical and lumbar lesions. The mechanism of injury was identified as low energy in 87.5%. (n=14) A high energy injury was noted in 12.5% (n = 2). The mean age of patients suffering from low energy injuries was 46.7 (± 12.6) years and that for patients with high energy injury was 42 (± 14.1) years. 68.7% (n=11) patients had neurological deficit with pain probably due to spinal instability at the time of presentation, while 32.2% (n=5) patients presented predominantly with pain due to spinal instability. Thus the incidence of neurological deficit in this series was 68.7% (n=11). The severity of deficit as assessed by Frankel grading was Grade B in 18.75% (three) patients, Grade C in 31.25% (five) and Grade D in 18.75% (three).

75% (n=12) patients had co-morbid medical conditions like hypertension, diabetes mellitus, poor pulmonary function, alcoholic liver disease and thyroid dysfunction. One patient (case no. 13) had to undergo a preoperative tracheostomy in view of anticipated difficult intubation due to poor visualization of the glottis during airway assessment. In the remaining patients, equipment to deal with difficult airway was kept standby during anesthesia.

**Figure 1:**
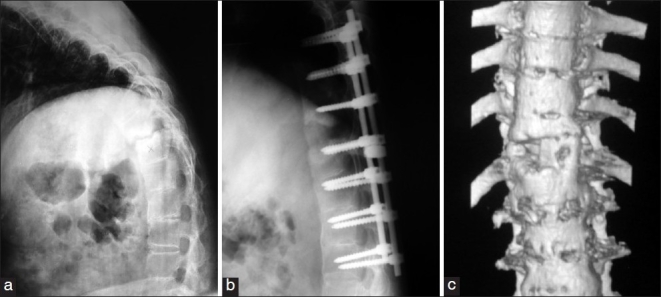
Case no. 11: (a) A pre-operative radiograph of D11-12 Andersson’s lesion in a male patient of 36 years age presenting with back pain due to spinal instability and neurological deficit. (b) A radiograph two years following D11-12 anterior decompression, bone grafting and D9-L3 posterior instrumented fusion. Patient had complete neurological recovery and pain relief after the surgery. (c) Three dimensional reconstruction CT scan image 2 years post surgery showing a sound D11-12 anterior fusion

**Figure 2:**
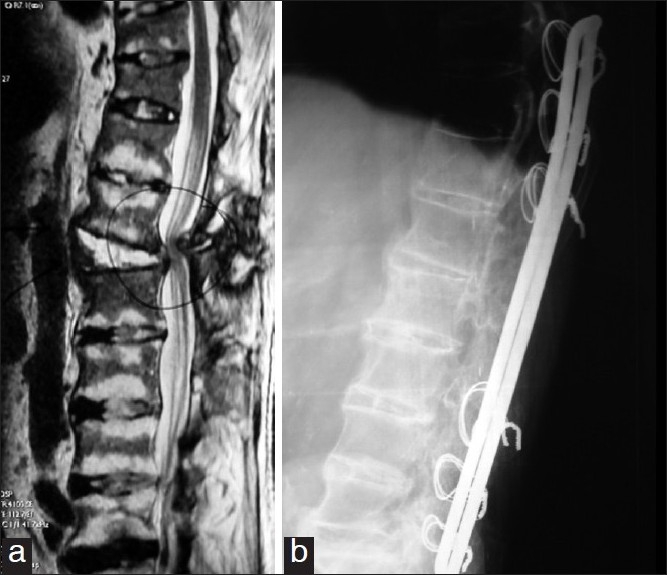
Case no. 7: (a) A pre-operative mid sagittal T2 weighted MR image of lumbar spine demonstrating a L1-2 Andersson’s lesion with significant anterior and posterior neural compression in a male patient of 60 years age presenting with cervical and lumbar spine injury, back pain due to spinal instability and neurological deficit. (b) A post-operative radiograph three years following L1-2 posterior decompression and D10-L5 posterior stabilization and instrumented fusion. Patient had relief of pain and complete improvement in the neurological status following the surgery

**Figure 3:**
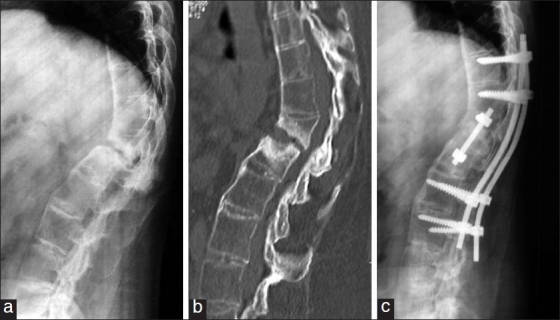
(a) Case no 10: A pre-operative radiograph of D12-L1 Andersson’s lesion in a male patient of 32 years age presenting with back pain due to instability. (b) A pre-operative mid sagittal CT scan reconstructed image demonstrating a D12-L1 Andersson’s lesion. (c) Case no. 10: Lateral radiograph at three years following D12-L1 anterior decompression, bone grafting, instrumented fusion and D10-L3 posterior stabilization and instrumented fusion. Patient had excellent pain relief following the surgery

All the patients were treated surgically. 87.5% (n=14) patients underwent decompression with instrumented fusion, while 12.5% (n=2) had decompression with uninstrumented fusion (case nos 2, 4); 18.8% (n=3) cases were treated by anterior surgery, 56.2% (n=9) by posterior surgery and 25% (n=4) by a combined approach; out of the two uninstrumented fusion, case no. 2 (D10-11 Andersson’s lesion) the spinal stability was considered adequate, while for case no. 4, (D11-12 Andersson’s lesion) the surgery for posterior instrumentation had to be avoided in view of poor skin condition, inadequate muscle cover and anticipated difficulty in achieving appropriate closure over the implant. 64.2% patients (Nine) were instrumented posteriorly (case nos. 1, 3, 6, 7, 8, 11, 13, 14, 15) [Figures 1a‐c, 2a‐b]; 21.4% patients (three) anteriorly (case nos. 5, 9, 16) while 14.3% patients (two) had antero-posterior instrumentation (case nos. 10, 12) [Figures 3a‐c]. Bone grafting was carried out in all the patients. The average intra-operative blood loss was 765.6 (± 472.5) ml. The average duration of the surgery was approximately 3.84 (± 1.14) hours [Table 1].

The mean patient follow-up was 54.5 (Range 18-154) months excluding a single mortality (case no. 16) in the post-surgical period due to medical complications. Of the 10 patients with neurological deficit who could be followed up, post surgical improvement in neurological status was observed in 90% (nine), while no significant recovery could be documented in one patient (case no. 3). Frankel grading improved from C to E in 31.25% (five) patients, D to E in 12.5% (two) and B to D in 12.5% (two).

There was significant improvement in the Visual analogue score for pain from a pre-surgical median of 8 to post-surgical median of 2 (*P*=0.001). A suboptimal relief in the intensity of pain was observed in 18.7% (three) patients (case nos. 4, 8, 14). Deformity correction could be attempted in 31.25% patients (five). These patients had presented with progressive worsening of the deformity following the onset of pain due to instability, and hence spinal deformity was an important component of their complaints. Three patients (case nos. 7, 10, 11) had complete correction (about 20-40 degrees) to their pre fracture sagittal alignment, while another (case no. 6) had partial correction (about 20 degrees). In one of the patients (case no. 8), only about 10 degrees of deformity correction could be achieved, resulting in major residual kyphotic deformity. Although the improvement in severity of the deformity was noted on the basis of clinical and radiological features, a detailed quantitative analysis of the deformity correction was not a part of this study.

At the time of this study, 68.7% (11) patients had returned to pre-injury functional status, with no major spine related complaints or evidence of clinical or radiological deterioration, while 25% (four) patients had mild to severe symptoms. Clinical and radiological evidence of fusion was present in 68.7% (n=11) patients. Features of pseudoarthrosis were noted in 12.5% (n=2) patients (case nos. 4, 8) at the final follow-up. Of these, case 4 had moderate pain with no progression of the deformity, which could be managed conservatively; while case 8 experienced worsening in the intensity of pain and deformity and was offered revision surgery. On the other hand, 12.5% (two) patients (case nos. 13, 14) had radiological evidence of inadequate fusion of which case 13 had minimal complaints while case 14 had moderate pain. Both the cases had no evidence of deformity progression and could be managed conservatively.

The early complications included intraoperative dural tears in two patients (case nos. 7, 8) and a sub optimal correction of a C1C2 rotatory subluxation in one patient (case no. 6). Both the patients with dural tears had a posterior approach taken for the surgery, with significant dural adhesions to the calcified ligamentum flavum, and could be managed by primary repair of the tear and appropriate postoperative measures. Appearance of significant anterior column defect following reduction by a posterior approach was observed in 18.8% (three) patients (case nos. 8, 11, 13). These patients were offered an anterior surgery to tackle the void, of which one (case no. 11) underwent the procedure at the same stage, since the problem was anticipated pre-operatively. There was one case of mortality (case no. 15) due to medical complications a week following surgery.

The late complications were observed in 25% (n=4) patients; case 13 with inadequate radiological fusion but minimal complaints requiring no additional measures, case 14 had inadequate fusion with moderate pain which could be managed conservatively. Case 4 had pseudoarthrosis with moderate pain requiring only conservative measures and case 8 had major problems, in the form of increased back pain and progressive deformity. This patient had a lower dorsal fracture, which was managed with a posterior instrumented fusion, but finally developed a pseudoarthrosis. This could be attributed to the lack of anterior surgery to augment the posterior surgery, which was necessary to achieve a three-column fusion.

## DISCUSSION

The ankylosed spine is susceptible to develop unstable lesions either due to vertebral fractures or an Andersson’s lesion (spondylodiscitis).^5^–^8^,^16^–^18^ There was a higher incidence of affected males 93.8% (15) than females (1), which is in accordance with the literature.^5^,^6^,^9^,^10^,^13^,^14^,^23^ A long lever arm resulting from a fused spine in association with severe osteoporosis makes the spine vulnerable to the most trivial injuries.^5^–^8^ 87.5% (14) patients had low energy trauma in the present series also. The most commonly associated symptom was chronic mechanical progressive back pain, most probably as a consequence of instability resulting from occult fractures or pseudoarthrosis involving the spine.^4^,^10^ Acute on chronic deterioration in the intensity of back pain, with or without a progressive deformity, may also indicate spinal instability.

An uncommon mode of presentation, in the form of acute spinal instability with neurological deficit, was seen in only 12.5% (two) patients with high energy trauma.^4^,^6^,^7^,^10^–^15^ A significant feature of these unstable lesions is the presence of associated neurological deficit.^6^,^15^,^23^ Our 11 (68.7%) patients presented with symptoms and signs suggestive of neural deficit.

A very high index of suspicion is necessary to arrive at this diagnosis in view of the occult nature of these lesions, pre-existing pain, deformity and few features suggesting the presence of a spinal fracture or spondylodiscitis in majority of the cases. In addition to plain radiography, the patients should be screened by magnetic resonance imaging and computed tomography.^4^ In addition, a CT-guided biopsy and Leukocyte-labeled bone scan may provide additional information.

Surgical intervention is indicated in presence of a two/ three-column injury, neurological deficit, a progressive deformity and significant pain due to instability.^20^–^22^ Complications associated with these fractures are not uncommon.^24^,^25^ The presence of medical co-morbidities and anesthetic risks may make the management complex. Difficulties in endotracheal intubation and positioning along with a compromised pulmonary function add to the perioperative risks. Poor muscle mass and skin condition, osteoporotic skeleton, dural adhesions, extensive blood loss, associated neurological deficit and problems in wound healing can complicate the intraoperative and the postoperative course.^5^–^8^ Extensive dural adhesions to the calcified ligamentum flavum is another common problem encountered, and this was evident in two of our patients in whom posterior exposure resulted in dural tears and defects.^4^

The principles of surgical management include neural decompression and an attempt to achieve global fusion with bone grafting and instrumentation whenever necessary. Indications for a combined anterior-posterior approach in a three-column injury are^21^–^25^ presence of kyphosis with an anterior/middle column defect. The surgical procedure here includes decompression, posterior instrumentation with fusion, followed by anterior decompression with fusion and bone grafting, either as a single stage or two-staged procedure.

Indications for only posterior approach in a three-column injury are^21^–^23^ presence of kyphosis with no significant anterior defect even after correction of the deformity. The surgical procedure here includes decompression, instrumented fusion with bone grafting and wherever required, a posterior column shortening procedure for deformity correction. The posterior approach, when used along with a posterior column shortening procedure, could help achieve significant correction of kyphosis and a global fusion, with minimal surgical morbidity. Indications for anterior approach are predominantly anterior lesions with a defect, with no major kyphosis, and a relatively intact posterior column. The procedure includes anterior decompression with instrumented fusion and bone grafting.

Instrumentation was necessary to augment the fusion as was evident in 87.5% of our cases. In an ankylosed spine, long segment instrumentation is necessary to counteract the strong deforming cantilever forces and prevent an implant failure.^23^–^25^ The surgical procedure can be extended to correct the spinal deformities under appropriate circumstances. A significant neurological deficit is a relative contraindication to achieve a deformity correction in addition to the primary procedure.

## CONCLUSION

The possibility of spinal instability, either due to a fracture or spondylodiscitis, should be extensively explored in patients with an ankylosed spine, who present with symptoms compatible with spinal injury, even without a history of trauma. The incidence of neurological deficit in these patients is significant. Management is challenging in view of the tendency of these lesions to develop a pseudoarthrosis, existing co-morbidities, complex surgical and anesthetic problems and high functional demands due to young age of these patients. The selection of the surgical approach is based on nature of the lesion, its location, the spinal curvature and the presence of neurological deficit.

The surgical procedure includes decompression, stabilization and fusion of the spine in appropriate alignment. Optimum surgical treatment can rehabilitate these patients to lead a productive and a near normal life.
